# Effects of *Barranca yajiagengensis* Powder in the Diet of *Trachinotus ovatus* on the Growth Performance, Antioxidant Capacity, Immunity and Morphology of the Liver and Intestine

**DOI:** 10.3390/antiox11071220

**Published:** 2022-06-22

**Authors:** Wei Zhao, Xin Cui, Zi-Qiao Wang, Rong Yao, Meng-Die Chen, Bao-Yan Gao, Cheng-Wu Zhang, Jin Niu

**Affiliations:** 1 State Key Laboratory of Biocontrol, Guangdong Provincial Key Laboratory for Aquatic Economic Animals and Southern Marine Science and Engineering Guangdong Laboratory (Zhuhai), Institute of Aquatic Economic Animals, School of Life Sciences, Sun Yat-Sen University, Guangzhou 510275, China; zhaow66@mail.sysu.edu.cn (W.Z.); cuix27@mail2.sysu.edu.cn (X.C.); wangzq26@mail2.sysu.edu.cn (Z.-Q.W.); yaor5@mail2.sysu.edu.cn (R.Y.); chenmd8@mail2.sysu.edu.cn (M.-D.C.); 2 Department of Ecology, Institute of Hydrobiology, College of Life Science and Technology, Jinan University, Guangzhou 510632, China; gaobaoyan1211@jnu.edu.cn

**Keywords:** *Trachinotus ovatus*, *Barranca yajiagengensis*, antioxidant ability, antibacterial ability, immunity, histomorphology

## Abstract

*Barranca yajiagengensis*, a novel filamentous microalga, can accumulate lutein under high-light and low-nitrogen conditions. It is well known that lutein has antioxidant, anti-inflammatory and immune-modulating properties. The purpose of this study is to evaluate the effects of including lutein-rich *B. yajiagengensis* powder in the diet of *Trachinotus ovatus* on the growth performance, antioxidant capacity, immunity, liver, and intestinal morphology. For this aim, three experimental diets containing 0% (BY0), 1% (BY1), and 5% (BY5) *B. yajiagengensis* powder were formulated for six-week feeding trials. The results indicated that growth performance, feed utilization, and intestinal morphology were not affected by different diet treatments. Fish fed with the BY5 diet promoted antioxidant ability by activating the Nrf2-ARE signal pathway and enhancing antioxidant enzymes activities. Furthermore, the BY5 diet improved non-specific immunity and antibacterial ability by activating lysozymes and the complement system and increasing the nitric oxide (NO) content and total nitric oxide synthase activity. Dietary *B. yajiagengensis* supplementation improved the liver morphology and exerted hepatoprotective effects. Therefore, as a natural source of lutein, *B. yajiagengensis* has the potential as a safe and non-toxic immunostimulant for *T. ovatus*. A diet supplemented with 5% *B. yajiagengensis* is recommended to improve the growth, antioxidant capacity, immune response, and liver health of *T. ovatus*.

## 1. Introduction

The golden pompano (*Trachinotus ovatus*) is a marine fish widely distributed in China, Japan, Australia, and Southeast Asia. It has become one of the most economically important mariculture fish species in southern China because of its fast growth rate, high-quality flesh, wide salinity tolerance, increasing market demand, and considerable economic efficiency. In China, the annual production of *T. ovatus* in 2020 was 101,693 metric tons [1]. At present, inshore net pens and intensive breeding mode are widely used in the culture of *T. ovatus* in China and Southeast Asian. However, owing to the polluted inshore water and extreme weather (e.g., typhoon, high temperature, rainstorm), *T. ovatus* cultured in intensive modes are more susceptible to infectious diseases and experimental stresses. Antibiotics are often used against such diseases, which not only does not result in satisfactory effects, but also threats food safety. Therefore, dietary supplementation with immunostimulants to enhance stress resistance and immunity is considered indispensable for the successful breeding of *T. ovatus*. In addition, such application can reduce the need of antibiotics and maintain sustainable development of aquaculture.

Microalgae contain a variety of bioactive molecules, such as carotenoids, polysaccharides, and fatty acids, therefore they have been widely used in health-care products, feeds, pharmaceuticals, and cosmetics based on their antioxidant, anti-inflammatory, anticancer, and immunomodulating activities [2]. In recent years, microalgae have also attracted great interest as a potential aquafeed immunostimulant. Many studies have demonstrated that dietary supplementation with microalgae improved the growth performance, antioxidant capacity, immunity, and stress resistance of fish [3,4,5,6]. Carotenoids of microalgae contribute to their antioxidant and coloring properties in aquaculture. Microalgae can produce a variety of carotenoids, including astaxanthin, lutein, β-carotene, canthaxanthin, fucoxanthin, and zeaxanthin [7]. Among such carotenoids, *Haematococcus pluvialis*-derived astaxanthin is considered to be an important and promising pigment in aquaculture because of its superior antioxidative properties. However, the cultivation of *H. pluvialis* is susceptible to microbial contamination, and the cost of harvesting and dewatering is expensive, which largely limit its application in aquaculture [8]. Commercial feeds supplemented with astaxanthin or *H. pluvialis* powder considerably increase feed cost and sales price. Therefore, it may be a good choice to find other cost-effective pigments or microalgae containing pigments to replace astaxanthin and *H. pluvialis* in aquatic feed.

Compared with astaxanthin, lutein in aquafeed also had beneficial effects on growth, antioxidant capacity, pigmentation, and immune response of aquatic animals [9,10,11]. At present, commercial natural lutein is mainly extracted from marigold. However, there are many problems such as slow growth rate of marigold, occupying the cultivated land for food crops, and excessive labor needed for harvesting. Microalgae are potential sources of natural lutein, such as *Chlorella pyrenoidosa*, *Scenedesmus almeriensis*, *Chlorococcum citroforme*, and *Tetracysis aplanosporum* [7,12]. Compared with marigold, the superiorities of lutein production from microalgae include high growth rate, low water demand, the ability to be cultivated on non-arable land, and the ability to be harvested all year [12]. Microalgae with high lutein accumulation are promising source of nature lutein, which has development and commercial potential.

The new species, *Barranca yajiagengensis*, was isolated from Yajiageng Red Rock Beach (Luding, Sichuan, China). This filamentous microalga belongs to Chaetophorales (Chlorophyta), which can accumulate a variety of high-value bioactive substances under different culture conditions, including linoleic acid, canthaxanthin and lutein [13]. The highest content of lutein reached 3.33 g/kg dry weight [13]. Compared with unicellular microalgae, filamentous microalgae have more advantages in industrialization because of their high biomass, high resistance to microbial pollution, and low-cost harvesting [14]. Therefore, as a natural source of lutein, *B. yajiagengensis* has the potential as an immunostimulant for aquatic animals. This study is aimed at determining the effects of dietary *B. yajiagengensis* powder on the growth, antioxidant capacity, immunity, and liver and intestinal health of *T. ovatus*. The results of the study can enrich the types of application of microalgae and the source of lutein in aquafeed.

## 2. Materials and Methods

### 2.1. B. yajiagengensis Cultivation and Diet Preparation

**Figure 1:**
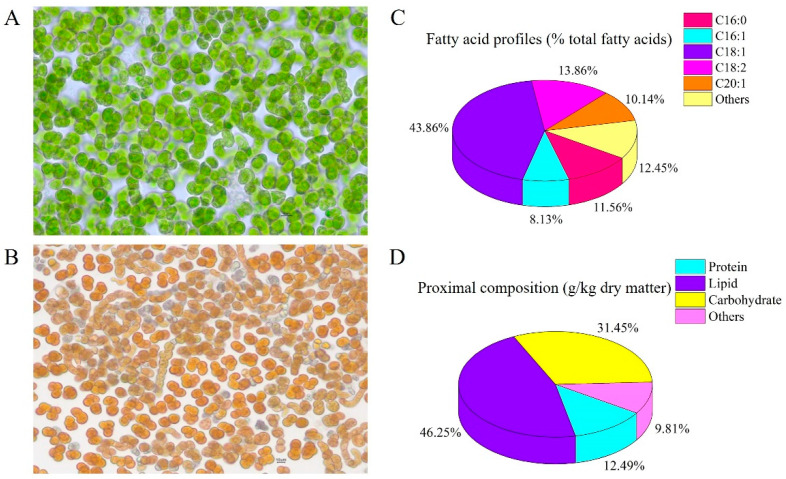
Cell morphology ((**A**), vegetative cell; (**B**), pigment accumulation cell), fatty acid profiles ((**C**), % total fatty acids) and proximal composition ((**D**), g/kg dry matter) of *Barranca yajiagengensis*.

The *B. yajiagengensis* were grown in a 160-L vertical flat-plate glass photobioreactor with a modified BG-11 medium [15] under the following cultivation conditions: 9 mmol L^−1^ NaNO_3_ as the initial nitrogen concentration and nitrogen source; continuous unilaterally light illumination of 300 μmol photons m^−2^ s^−1^; temperature was maintained at 25 ± 1 °C; aerated with 1% (*v*/*v*) CO_2_-enriched compressed air. On Day 15, *B. yajiagengensis* cells were collected by mesh filtration, and then freeze-dried by freezing dryer to obtain microalgae powder. The *B. yajiagengensis* powder contained 0.33% lutein and 4.4% linoleic acid (Figure 1).

Three isonitrogenous and isolipidic experimental diets were formulated to contain 0% (BY0), 1% (BY1), and 5% (BY5) *B. yajiagengensis* powders. The formula and nutrient levels of experimental diets were presented in Table 1. All ingredients were thoroughly ground using a grinder and then sifted through a 100 μm sieve to obtain a uniform sample. Based on the feed formula, all required ingredients were weighed and thoroughly mixed. Then, distilled water (300 g kg^−1^ dry matter) was added to the mixture. The prepared mixtures were transferred into a puffing apparatus (Institute of Chemical Engineering, South China University of Technology, Guangdong, China), and the final 2.5-mm-diameter puffed pellets were produced. The diets were dried in an air-conditioned room until the moisture level was below 10%, and then stored at −20 °C.

### 2.2. Fish and Feeding Trial

The nutritional feeding trial was conducted in Lingshui bay, Hainan, China. *T. ovatus* juveniles were obtained from a local commercial company and acclimatized in a floating sea cage (3.0 m × 3.0 m × 5.0 m) for 14 days. During the acclimation, fish were fed two times daily with the control diet. At the beginning of the trial, all fish were fasted for 24 h, and then 180 fish of similar size (7.06 ± 0.12 g) were selected and randomly assigned into 9 sea cages (1.0 m × 1.0 m × 1.5 m) at a density of 20 fish per cage. Each experimental diet was randomly applied to three cages. Fish were fed slowly by hand to apparent satiation twice daily (08:00 and 17:00) for 42 days. Feed consumption and the number and weight of dead fish were recorded every day.

### 2.3. Sampling Collection

The animal use protocol listed below has been reviewed and approved by the Institution Animal Care and Use Committee, Sun Yat-Sen University (approval code: SYSU-IACUC-2022-B0159). At the end of the trial, the feeding process stopped for 24 h and then fish were anesthetized with tricaine methanesulfonate (Sigma, St. Louis, MO, USA). All fish from each replication were counted and weighed to determine growth performance. Blood samples were collected from six fish per cage by a 1 mL syringe from the caudal vein, then stored overnight at 4 °C, and finally centrifuged (4 °C, 4000 r/min, 10 min) to collect the serum for analysis of hematological parameters and antioxidant enzyme activities. The liver and midgut of the aforementioned six fish were removed and washed with double distilled water (4 °C) on ice, and then rapidly frozen in liquid nitrogen for analysis of digestive enzymes, antioxidant enzymes, and gene expression. Finally, the liver and midgut samples from three fish per cage were removed and fixed in 4% paraformaldehyde for histological analysis.

### 2.4. Biochemical Analysis of B. yajiagengensis and Experimental Diets

The total lipids in the *B. yajiagengensis* powder were determined with the gravimetric method following the procedures described by Gao et al. (2016) [16]. The fatty acid profiles of *B. yajiagengensis* were analyzed by gas chromatograph (6890 N GC, Agilent Technologies, Palo Alto, CA, USA) according to the procedures designed by Zhang et al. (2018) [17]. Total carbohydrate content of *B. yajiagengensis* powder was measured by the phenolsulfuric acid method (Wang et al. 2019) [18]. Total protein of *B. yajiagengensis* powder was quantified by commercial assay kits (Sangon Biotech, Shanghai, China) based on the Lowry method. The lutein content in the *B. yajiagengensis* powder was measured according to Gao et al. (2022) [13].

Crude protein, crude lipid, moisture, and ash contents in experimental diets were measured following the method described by the Association of Official Analytical Chemists (AOAC, 1995) [19].

### 2.5. Enzyme Activity Assays

The midgut and liver samples were weighed and homogenized in ice-cold normal saline at a volume ratio of 1:9, and then centrifuged at 3000 r/min (4 °C) for 10 min to obtain the supernatant. The supernatant of liver and midgut tissues as well as serum were used for the determination of enzyme activity.

Antioxidant parameters from the lipid soluble antioxidant system, including superoxide dismutase (SOD, EC1.15.1.1), catalase (CAT, EC1.11.1.6), and glutathione peroxidase (GSH-PX, EC1.11.1.9), plus total antioxidant capacity (T-AOC) and malondialdehyde (MDA) content, were measured with kits NJBI (Nanjing Jiancheng Bioengineering Institute, Nanjing, China). Briefly, the supernatant of the liver sample was diluted 30 times and used for the determination of SOD activity at 37 °C, and the absorbance was measured at 530 nm. The supernatant of the liver sample was diluted eight times and used for the determination of CAT activity at 37 °C, and the absorbance was measured at 405 nm. The supernatant of the liver sample was used for the determination of GSH-PX activity at 37 °C, and the absorbance was measured at 412 nm. The serum SOD activity was measured at 37 °C, and the absorbance was measured at 530 nm.

The activities of digestive enzymes in the midgut, including amylase (AMS, EC3.2.1.1), pepsin (PEP, EC3.4.23.1), and lipase (LPS, EC3.1.1.3), were measured spectrophotometrically by kits from NJBI, China. Briefly, the supernatant of the midgut sample was diluted 20 times and used for the determination of AMS activity at 37 °C, and the absorbance was measured at 660 nm. The supernatant of the midgut sample was used for the determination of PEP activity at 37 °C, and the absorbance was measured at 660 nm. The supernatant of the midgut sample was used for the determination of LPS activity at 37 °C, and the absorbance was measured at 580 nm.

### 2.6. Immune-Related Parameters Assays

Immune-related parameters in the liver and serum were assayed using commercial kits (NJBI, Nanjing, China) following the provided instructions, including nitric oxide (NO) content and total nitric oxide synthase (TNOS, EC1.14.13.39) activity in the liver, and lysozyme (EC3.2.1.17) activity and complement 4 (C4) in serum.

### 2.7. Histological Observation

Midgut and liver samples were fixed in 4% paraformaldehyde, and then dehydrated in a graded series of ethanol (75%, 4 h; 85%, 2 h; 90%, 2 h; 95%, 1 h; 100%, 1 h) and embedded in paraffin wax. Sections (5 μm thick) of midgut and liver were obtained with a rotary microtome (RM2016, Leica, Wetzlar, Germany) and stained with hematoxylin and eosin. Finally, the sections were observed and photographed using an optical microscope (Leica DMLB, Wetzlar, Germany).

### 2.8. Serum Parameters Assays

Serum biochemical parameters, including triglyceride (TG), glucose (GLU), low-density lipoprotein cholesterol (LDL-C), and high-density lipoprotein cholesterol (HDL-C) were measured by the colorimetric method performed using an automatic biochemical analyzer Chemray 240 (Rayto Life Science Co., Ltd., Shenzhen, China) and corresponding commercial kits (Huili Biotech Co., Ltd., Changchun, China).

### 2.9. RNA Extraction and Gene Expression Analysis

The total RNA extraction and quantitative reverse transcription polymerase chain reaction (qRT-PCR) were performed according to Zhao et al. (2020) [14]. Briefly, total RNA from the liver in each cage was isolated using a reagent kit (TaKaRa, Dalian, China) following the manufacturer’s protocol. RNA sample quality was assessed by 1% agarose gel electrophoresis and quantified by spectrophotometric analysis (OD260/280) using the Nanodrop Lite (Thermo Scientific, Waltham, MA, USA). Then, the total RNA samples were diluted to the same concentration with diethylpyrocarbonate treated water for normalization. Subsequently, cDNA was synthesized using a PrimeScript RT Reagent kit with gDNA Eraser (TaKaRa, Dalian, China) according to the provided instructions. qRT-PCR analysis was performed on a LightCycler 480 Real-Time System (Roche Applied Science, Basel, Switzerland) with SYBR^®^ Premix ExTaq™ II (TaKaRa, Dalian, China).

To evaluate the immunomodulatory effects of *B. yajiagengensis*, the following gene was selected: antioxidant factors, including NF-E2-related nuclear factor 2 (*Nrf2*), Kelch-like-ECH-associated protein 1 (*Keap1*), glutathione reductase (*GR*), superoxide dismutase (*Mn-SOD*), and haemoxygenase-1 (*HO-1*); pro-inflammatory cytokines, including interleukin 1β (*IL-1β*) and interleukin 8 *(IL-8*); anti-inflammatory cytokines, including interleukin 10 (*IL-10*) and transforming growth factor β1 (*TGF-β1*); and immune-related factors, including c-type lysozyme (*C-Lyz*) and *C4*. Primers used for qRT-PCR were previously published (Zhao et al., 2020) [14]. Data were normalized using the β-actin. The relative expression levels of target genes were calculated based on the 2^−ΔΔCT^ method [20].

### 2.10. Statistical Analysis

The specific growth ratio (SGR), survival rate (SR), weight gain rate (WGR), and feed conversion ratio (FCR) were calculated according to Zhao et al. (2022) [3].

All data were presented as the means ± standard error (SE) and analyzed using SPSS 20.0 statistical software (SPSS, Chicago, IL, USA). All data were checked for normality with the Kolmogorov-Smirnov test and homogeneity with the Levene’s test. The differences in data were assessed by using one-way analysis of variance (ANOVA) followed by Tukey test. Differences with *p* < 0.05 were considered to be statistically significant.

## 3. Results

### 3.1. Biological Performance

**Figure 2:**
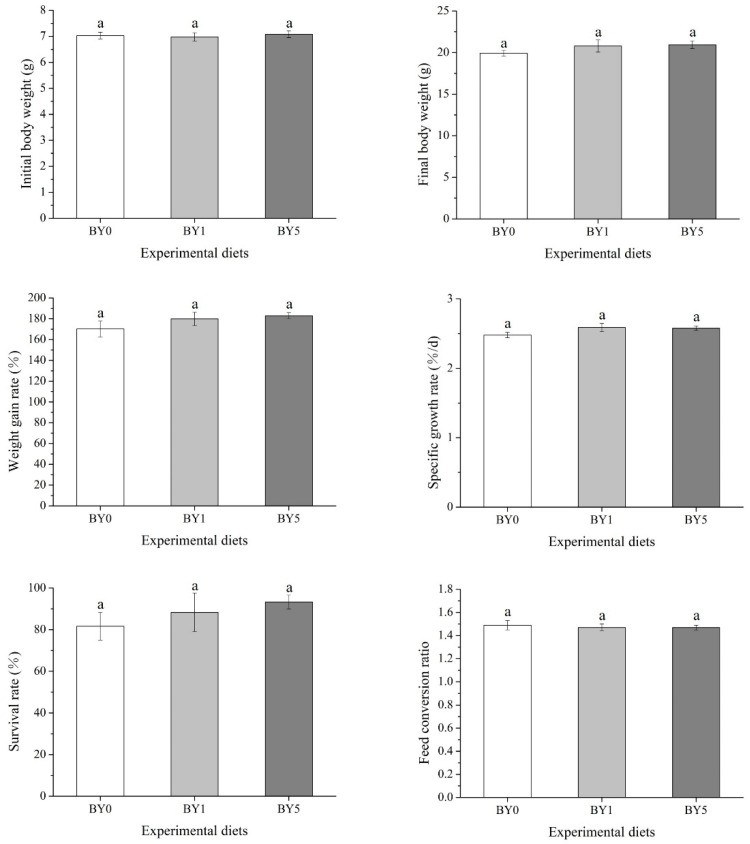
Effects of dietary *Barranca yajiagengensis* powder supplementation on growth performance and feed utilization of *T. ovatus* after 42 day feeding trial. The same letters indicate no significant differences (*p* > 0.05).

The results of biological performance of fish fed with experimental diets were summarized in Figure 2. The results showed that final body weight, SGR, WGR, FCR and SR were not affected by different diet treatments (*p* > 0.05).

### 3.2. Serum Biochemical Parameters

The results obtained from the determination of serum biochemical parameters of fish fed with experimental diets were presented in Table 2. The results showed that the contents of TG, GLU, LDL-C, HDL-C, and HDL-C/LDL-C ratio in different diet treatments did not show a significant difference (*p* > 0.05).

### 3.3. Antioxidant Parameters

**Figure 3:**
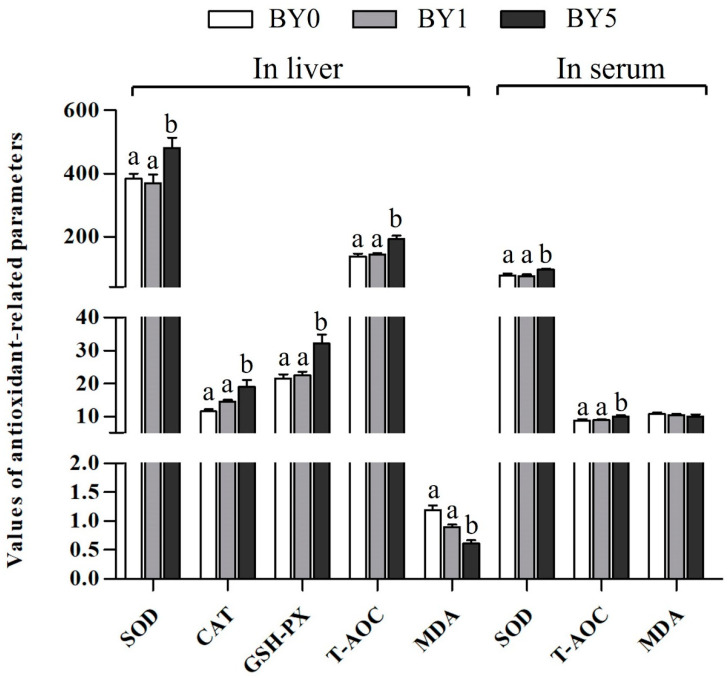
Antioxidant-related parameters in liver and serum of *T. ovatus* fed experimental diets. Values are presented as mean ± SE (*n* = 18). The small letters indicate significant differences at *p* < 0.05. SOD, superoxide dismutase (U mg protein^−1^ in liver; U mL^−1^ in serum); CAT, catalase (U mg protein^−1^); GSH-PX, glutathione peroxidase (U mg protein^−1^); T-AOC, total antioxidant capacity (mmol g protein^−1^ in liver; mmol L^−1^ in serum); MDA, malondialdehyde (nmol mg protein^−1^ in liver; nmol mL^−1^ in serum).

As shown in Figure 3, the T-AOC and activities of CAT, GSH-PX, and SOD in the liver of the BY5 diet treatment increased significantly compared to the BY0 and BY1 diet treatments, while MDA content in the liver decreased significantly in the BY5 diet treatment (*p* < 0.05). Serum SOD activity and T-AOC in the BY5 diet treatment was significantly higher than that of the BY0 and BY1 diet treatments (*p* < 0.05). However, serum MDA contents were not affected by different diet treatments (*p* > 0.05).

### 3.4. Immune Biochemical Parameters

**Figure 4:**
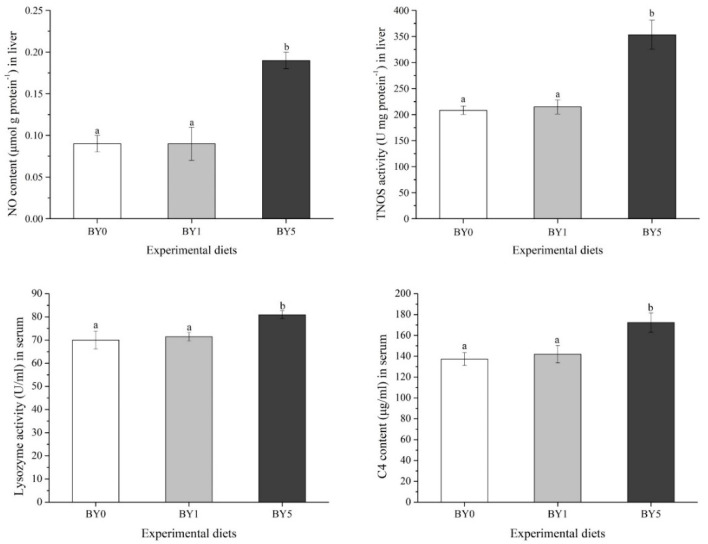
Immune-related parameters in liver and serum of *T. ovatus* fed experimental diets. Values are presented as mean ± SE (*n* = 18). The small letters indicate significant differences at *p* < 0.05.

As shown in Figure 4, the NO content and TNOS activity in the liver as well as lysozyme activity and C4 content in the serum of the BY5 diet treatment increased significantly compared to the BY0 and BY1 diet treatments (*p* < 0.05).

### 3.5. Morphology of Liver and Midgut and Activities of Digestive Enzymes in Midgut

**Figure 5:**
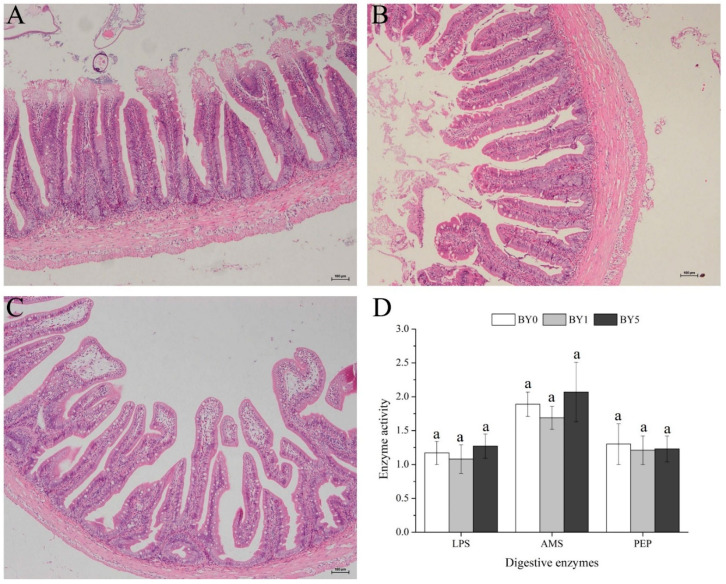
Midgut morphology of *T. ovatus* fed with BY0 (**A**), BY1 (**B**) and BY5 (**C**) diets, and digestive enzymes activities in the midgut of fish fed experimental diets (**D**). Values are presented as mean ± SE (*n* = 18). The same letters indicate no significant differences (*p* > 0.05). LPS, lipase (U g protein^−1^); AMS, amylase (U mg protein^−1^); PEP, pepsin (U mg protein^−1^). Scale bar: 100 μm.

As shown in Figure 5, no obvious histological alterations were observed in the midgut among all diet treatments. In addition, the activities of digestive enzymes in the midgut, including LPS, AMS, and PEP, were not affected by different diet treatments (*p* < 0.05).

**Figure 6:**
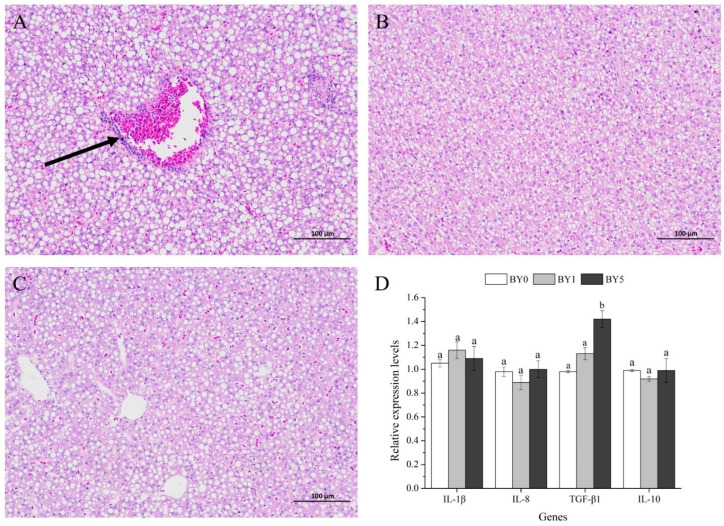
Liver morphology of *T. ovatus* fed with BY0 (**A**), BY1 (**B**), and BY5 (**C**) diets, and relative expression levels of inflammation-related genes in the liver of fish fed experimental diets (**D**). The black arrow indicates the infiltration of inflammatory cells. Values are presented as mean ± SE (*n* = 18). The small letters indicate significant differences at *p* < 0.05. IL-1β, interleukin 1β; IL-8, interleukin 8; TGF-β1, transforming growth factor β1; IL-10, interleukin 10.

As shown in Figure 6A–C, the liver of fish fed with diet supplemented with *B. yajiagengensis* showed a healthy morphology, while mild inflammatory cell infiltration was observed in the liver from the BY0 diet.

### 3.6. Expression Analysis of Antioxidant-Related and Immune-Related Genes in Liver

Fish fed with the BY5 diet showed significantly higher mRNA levels of *TGF-β1* (*p* < 0.05), while the mRNA levels of *IL-1β*, *IL-8*, and *IL-10* were not affected by different diet treatments (*p* > 0.05) (Figure 6D).

**Figure 7:**
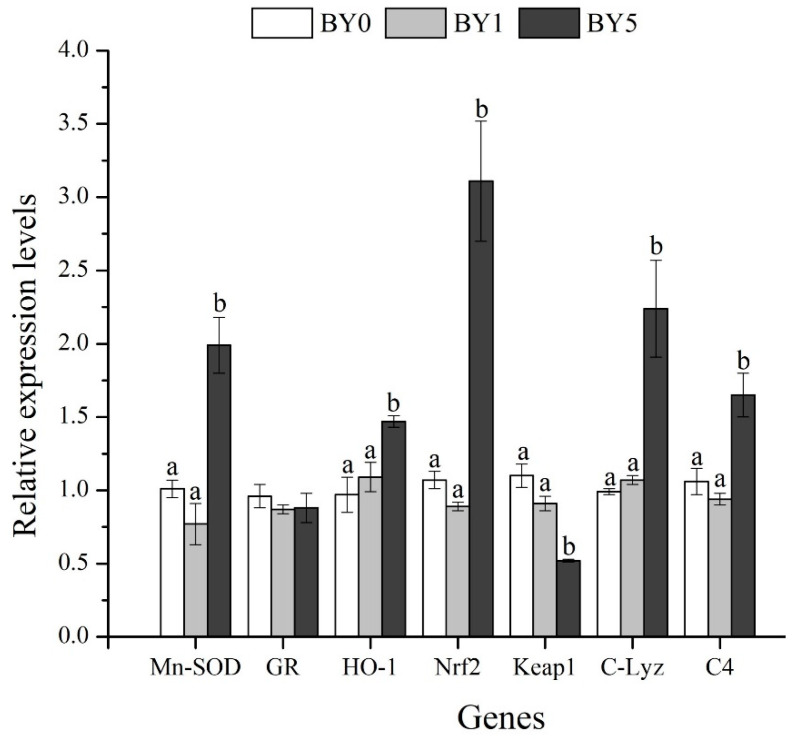
Relative expression levels of antioxidant-related and immune-related genes in the liver of *T. ovatus* fed experimental diets. Values are presented as mean ± SE (*n* = 18). The small letters indicate significant differences at *p* < 0.05. Mn-SOD, manganese superoxide dismutase; GR, glutathione reductase; HO-1, haeme oxygenase-1; Nrf2, NF-E2-related nuclear factor 2; Keap1, Kelch-like-ECH-associated protein 1; C-Lyz, c-type lysozyme; C4, complement 4.

Compared with the BY0 and BY1 diet treatments, the mRNA levels of *GR*, *Nrf2*, *Mn-SOD*, *HO-1*, *C-Lyz*, and *C4* were significantly enhanced in the BY5 diet treatment (*p* < 0.05), while the mRNA level of *Keap1* decreased significantly in the BY5 diet treatment (*p* < 0.05) (Figure 7).

## 4. Discussion

The findings obtained in the current study showed that dietary *B. yajiagengensis* powder supplementation had no significant effects on growth performance and feed utilization of *T. ovatus*. The results were consistent with similar studies on the effect of lutein on the growth and feed utilization of fish, such as *Amphiprion ocellaris* [21], *Carassius auratus* [11], and *Larimichthys croceus* [22]. Digestive enzyme activity is closely related to the digestion and absorption of nutrients and the growth of aquatic animals [14,23]. In this study, the activities of digestive enzymes (LPS, AMS, and PEP) in the midgut were not affected by different diet treatments, which was consistent with growth performance. The results demonstrated that dietary *B. yajiagengensis* powder supplementation did not accelerate the digestion process to improve the feed utilization and growth of *T. ovatus*. However, Ettefaghdoost and Haghighi (2021) [10] indicated that adding 50–200 mg/kg of lutein to the diet enhanced growth, feed utilization, and digestive enzyme activities (LPS, AMS, protease) of *Macrobrachium nipponense*. Lutein acts as a mediator in metabolism, which accelerates the digestion process and ultimately promotes growth performance [10]. Comparison of results from previous studies with those of the current study revealed that there are species differences in the effects of lutein on growth performance and feed utilization of aquatic animals.

The observation of tissue morphology can intuitively show the health status of fish. In the current study, liver morphological examination revealed mild inflammatory cell infiltration in fish fed with BY0 diet, which was largely due to the influence of environmental stress. Fish cultured in offshore cages are more vulnerable to various environmental stressors (e.g., typhoons, rainstorm, hypoxia, temperature, pollutants), which may result in peroxidation and inflammatory response in liver. The liver of fish fed with diets supplemented with *B. yajiagengensis* showed a healthy morphology, which demonstrated that *B. yajiagengensis* exerted hepatoprotective effects. In fish, inflammatory response is regulated by pro-inflammatory cytokines and anti-inflammatory cytokines. Pro-inflammatory cytokines, such as IL-1β and IL-8, are initially activated during inflammatory responses; however, subsequent release of anti-inflammatory cytokines, such as IL-10 and TGF-β1, can robustly inhibit this effect, thereby counteracting the hyperactivity of immune responses triggered by pro-inflammatory cytokines [24,25]. Therefore, to further clarify the hepatoprotective mechanism of *B. yajiagengensis*, the current study determined the effects of *B. yajiagengensis* on the mRNA levels of inflammation-related genes. The findings obtained in the current study showed that fish fed with the BY5 diet showed significantly higher mRNA level of *TGF-β1*. The current results indicated that *B. yajiagengensis* has anti-inflammatory properties and potently suppresses inflammatory responses in the liver, which may be mainly attributed to the lutein contained in *B. yajiagengensis*. Lutein exhibits anti-inflammatory activity by decreasing inflammatory proteins and pro-inflammatory cytokines, and increasing anti-inflammatory cytokines [26,27,28]. In addition, midgut morphological examination showed no obvious pathological alterations among all diet treatments. The results of morphological observation confirmed that *B. yajiagengensis* is a safe and non-toxic feed additive and exerted hepatoprotective effects by upregulating the expression of anti-inflammatory cytokines.

The Nrf2-ARE pathway plays a significant role in protecting cells from oxidative stress by removing reactive oxidants [29]. Nrf2 and Keap1 are two key transcription factors in the Nrf2-ARE pathway. Keap1, as a repressor of Nrf2, combines with Nrf2 to form an Nrf2-Keap1 complex under normal homeostatic conditions, which prevents the translocation of Nrf2 from cytoplasm to nucleus and promotes Nrf2 degradation [30]. Once stimulated by oxidative stress, Nrf2 dissociates from Keap1 and subsequently binds to antioxidant-responsive elements in the nucleus to induce transcription of antioxidant enzyme genes, such as *GR*, *HO-1*, and *Mn-SOD* [29]. Therefore, to evaluate the antioxidant properties of microalgae, the current study determined the effects of *B. yajiagengensis* on the mRNA levels of genes related to the Nrf2-ARE pathway. The results showed that fish fed with the BY5 diet upregulated *Nrf2* mRNA levels and downregulated *Keap1* mRNA levels in the liver. Further analysis showed that the mRNA levels of *Mn-SOD* and *HO-1* were also significantly upregulated in the BY5 diet treatment. Similarly, a previous study reported that lutein exerts protection against oxidative stress by upregulating *Nrf2* and *HO-1* expression [27]. The findings obtained in the current study indicated that 5% *B. yajiagengensis* powder promoted the antioxidant ability of *T. ovatus* by activating the Nrf2-ARE signal pathway.

It is well known that lipid-soluble antioxidant systems, such as SOD, CAT, and GSH-PX, protect the cell against free radical-induced oxidation. As the first line of antioxidant defense in cells to resist the toxicity caused by free radicals, SOD and CAT can prevent the formation of free radicals and protect cell membrane lipids from peroxidation [10]. SOD can convert superoxide (^•^O_2_^−^) to H_2_O_2_ via the dismutation of superoxide, which helps to maintain the redox balance in cells and protect cells from peroxidative damage. CAT is an oxidoreductase enzyme that converts H_2_O_2_ into water and molecular oxygen. GSH-PX is a peroxidase with strong reducibility, which catalyzes the reduction of H_2_O_2_ and lipid hydroperoxides, protecting the liver from oxidative damage. The increased enzyme activity is caused by the increased synthesis of enzyme protein, which largely relies on its gene transcription and translation [31]. Therefore, to further confirm whether the elevated Nrf2 mRNA level contributes to increasing the activities of its downstream antioxidant enzymes, the current study measured the effects of *B. yajiagengensis* on lipid-soluble antioxidant system activities that protect polyunsaturated fatty acids (PUFAs) in biomembranes. In the current study, fish fed with the BY5 diet boosted the lipid-soluble antioxidant systems. Similar results have also been reported in which dietary lutein improved the antioxidant potential of mice by increasing the activities of SOD, CAT, and GSH-PX in the liver [32]. In addition, T-AOC and MDA are indicators for evaluating the balance between oxidants and antioxidant factors. T-AOC directly reflects the antioxidant capacity of fish, while the MDA level indirectly reflects the damage degree of cells attacked by free radicals [33]. According to the study results, T-AOC increased significantly in the liver and serum of fish fed with the BY5 diet, while MDA decreased significantly in the liver. The aforementioned results suggested that 5% *B. yajiagengensis* boosted the lipid-soluble antioxidant systems, protecting PUFA in dietary oil and protecting PUFAs in biomembranes. Based on the results of antioxidant parameters determined in this study, the dietary supplement of 5% *B. yajiagengensis* powder improved the antioxidant capacity of *T. ovatus* by activating the Nrf2-ARE pathway and enhancing lipid-soluble antioxidant systems including SOD, CAT, and GSH-PX and protected lipids from peroxidation.

Compared with pond- and factory-farming models, offshore cage culture is often affected by environmental stressors and increases the risk of pathogen infection in fish. Therefore, antibacterial ability is an important indicator to evaluate the effect of immunostimulants on fish immunity. NO, as a gaseous signaling molecule, is involved in the regulation of neuronal transmission and anti-inflammatory, anti-tumor, and antibacterial activities [34]. NO can react with free radical superoxide to generate active substances including nitrogen dioxide, dinitrogen trioxide, and peroxynitrite, which cause severe nitrosation and oxidative stress to bacteria, eventually destroying cell membranes and causing cell dysfunction of bacteria [35]. Therefore, NO is considered to be an effective bactericidal agent that kills broad-spectrum bacteria, especially drug-resistant ones [36]. NO is produced by NOS-catalyzed reaction of *_L_*-arginine with molecular oxygen [37]. Therefore, there was a positive correlation between NO content and NOS activity. NO and NOS are considered to be antibacterial molecules against pathogen infection in aquatic animals [38,39]. In this study, TNOS activity and NO content increased significantly in liver of fish fed with the BY5 diet. The findings obtained in the current study indicated that 5% *B. yajiagengensis* powder promoted the defense ability of *T. ovatus* against pathogenic infection.

The non-specific immune system of teleost fish plays a more important role in resisting pathogen invasion and secondary damage than that of other vertebrates [40]. In fish, the complement system plays a central role in the immune response of fish against pathogen infection, and is responsible for the clearance of cellular debris, apoptotic cells, and foreign invaders [41]. In addition, it can bind to specific sites on the surface of phagocytes to promote phagocytosis [42]. Lysozyme, as an antibacterial molecule, can cleave bacteria by hydrolyzing the β-1,4 glycosidic bond of peptidoglycan layer of the bacterial cell wall [43,44]. Previous studies have shown that Lyz and the complement system can act synergistically in bactericidal processes [45,46]. In this study, fish fed with the BY5 diet upregulated the mRNA levels of *C-Lyz* and *C4* in the liver and increased the C4 content and Lyz activity in the serum. Similarly, dietary astaxanthin supplementation improved the immunity of *Pseudosciaena crocea* by increasing lysozyme activity and complement content in the serum [47]. Zhao et al. (2021) [48] also demonstrated that *T. ovatus* fed with astaxanthin-rich *Haematococcus pluvialis* upregulated the mRNA expression of *C-Lyz* and *C4* in the liver. The current results suggested that 5% *B. yajiagengensis* could improve the non-specific immune response and antibacterial ability of *T. ovatus* by activating Lyz and the complement system.

## 5. Conclusions

This study demonstrated that *Barranca yajiagengensis* exerted beneficial effects in *T. ovatus*. The diet supplementation of 5% *B. yajiagengensis* powder promoted antioxidant responses of *T. ovatus* by activating the Nrf2-ARE signal pathway and enhanced the lipid-soluble antioxidant system, improving non-specific immunity and antibacterial ability by activating the complement system and Lyz, and increasing the NO content and TNOS activity. In addition, dietary *B. yajiagengensis* supplementation improved the liver morphology and exerted hepatoprotective effects. Therefore, as a natural source of lutein, *B. yajiagengensis* has potential as a safe and non-toxic immunostimulant for *T. ovatus*. The application of *B. yajiagengensis* can enrich the source of lutein in aquafeed.

## Figures and Tables

**Table 1:** Composition and nutrient levels of the experimental diets (% dry matter).

Ingredients	BY0	BY1	BY5
Fish meal	45	45	45
Soybean meal	16.3	16	15.4
Wheat flour	20	20	20
Beer yeast	3	3	3
Microcrystalline Cellulose	4	3.7	2.1
Fish oil	7	6.6	4.8
Soybean lecithin	1	1	1
Ca(H_2_PO_4_)_2_	1	1	1
Vitamin premix ^a^	1	1	1
Mineral premix ^b^	1	1	1
Choline	0.5	0.5	0.5
Vitamin C	0.2	0.2	0.2
*Barranca yajiagengensis*	0	1	5
Total	100	100	100
Nutrient levels ^c^			
Crude lipid	12.31	12.22	12.55
Crude protein	42.27	42.26	41.45
Ash	10.31	10.38	10.75
Moisture	8.92	9.63	9.16
Lutein (mg kg^−1^ dry matter)	-	31.20	156.18

^a^ Vitamin premix provides the following per kg of diet: VB_1_ 25 mg, VB_2_ 45 mg, pyridoxine HCl 20 mg, VB_12_ 0.1 mg, VK_3_ 10 mg, inositol 800 mg, pantothenic acid 60 mg, niacin acid 200 mg, folic acid 20 mg, biotin 1.20 mg, retinal acetate 32 mg, cholecalciferol 5 mg, α-tocopherolα 120 mg, ascorbic acid 2000 mg, choline chloride 2500 mg, ethoxyquin 150 mg, wheat middling 14.012 g. ^b^ Mineral premix provides the following per kg of diet: NaF 2 mg, KI 0.8 mg, CoCl_2_·6H_2_O 50 mg, CuSO_4_·5H_2_O 10 mg, FeSO_4_·H_2_O 80 mg, ZnSO_4_·H_2_O 50 mg, MnSO_4_·H_2_O 60 mg, MgSO_4_·7H_2_O 1200 mg, Ca(H_2_PO_4_)_2_·H_2_O 3000 mg, NaCl 100 mg, zeolite 15.447 g. ^c^ Measured values.

**Table 2:** Serum parameters of *Trachinotus ovatus* fed experimental diets for 42 days.

Items	BY0	BY1	BY5
TG	2.22 ± 0.15	2.44 ± 0.13	2.30 ± 0.18
GLU	3.89 ± 0.50	4.11 ± 0.30	3.89 ± 0.37
LDL-C	3.14 ± 0.12	3.04 ± 0.10	3.26 ± 0.05
HDL-C	1.05 ± 0.04	1.05 ± 0.05	1.17 ± 0.04
HDL-C/LDL-C	0.34 ± 0.02	0.34 ± 0.01	0.36 ± 0.01

Values are presented as mean ± SE (*n* = 18). TG, triglyceride (mmol L^−1^); GLU, glucose (mmol L^−1^); LDL-C, low-density lipoprotein cholesterol (mmol L^−1^); HDL-C, high-density lipoprotein cholesterol (mmol L^−1^).

## Data Availability

The data presented in this study are available in the article.
